# Binary Biopolymer Blends: Influence of Mixing Procedure on Mechanical Properties of Polymer Thin Films

**DOI:** 10.3390/ma19163485

**Published:** 2026-08-18

**Authors:** Aleksandra Nešić, Branka Pilić

**Affiliations:** Faculty of Technology Novi Sad, University of Novi Sad, Bulevar Cara Lazara 1, 21000 Novi Sad, Serbia; brapi@uns.ac.rs

**Keywords:** polylactic acid, PBAT, PBS, biopolymer blends, double processing, PEG plasticizer, thin films, mechanical properties, DSC, contact angle

## Abstract

**Highlights:**

- Comprehensive statistical analysis showed that double processing of PLA/PBS and PLA/PBAT significantly improved the mechanical properties relative to single-processed blends and to blends containing PEG 4000 or PEG 20000.
- Addition of PEG 4000 and PEG 20000 at 1–5 wt% modestly improves tensile stress but does not replicate the ductility gains achieved by reprocessing.
- DSC analysis reveals Tg depression and altered crystallisation behaviour in double-processed blends, consistent with improved phase compatibility.
- Contact angle measurements indicate comparable surface wettability across series, suggesting mechanical differences arise from bulk morphology rather than surface changes.
- FTIR spectroscopy confirms the absence of chemical reactions between the blend components; morphological refinement of the dispersed phase is therefore proposed as the most probable mechanism.

**Abstract:**

Polylactic acid (PLA) is one of the most promising bio-based and biodegradable polymers, yet its inherent brittleness limits its application in flexible film products. This study compares three mixing strategies, applied as four sample series, to improve the mechanical performance of PLA-based binary blends: (1) single-pass melt blending with poly(butylene adipate-co-terephthalate) (PBAT) or poly(butylene succinate) (PBS) at 10, 20, and 30 wt%; (2) addition of poly(ethylene glycol) (PEG 4000 or PEG 20000) as a plasticizer/compatibilizer at 1, 3, and 5 wt%; (3) double melt processing of PLA/PBAT and PLA/PBS blends. Thin films were characterised by tensile testing, differential scanning calorimetry (DSC), FTIR, SEM and contact angle measurements. Double processing emerged as the most effective approach, yielding elongation at break values up to approximately 137% for 70PLA/30PBAT blends, compared to 16.9% for the equivalent single-processed samples. Relative to the single-processed controls, double processing raised elongation at break by approximately 712% for 70PLA/30PBAT and 1201% for 70PLA/30PBS, and by 98% (80PLA/20PBAT), 175% (80PLA/20PBS) and 278% (90PLA/10PBAT); the latter three increases were statistically significant (*p* < 0.05). By contrast, PEG addition changed maximum stress by at most about 18% and never raised elongation at maximum stress above 6%. Two-way ANOVA confirmed that blend ratio was a significant factor for maximum stress (*p* < 0.001) whereas PEG molecular weight was not (*p* > 0.10). PEG addition produced moderate improvements in tensile stress, but did not replicate the ductility enhancement observed after reprocessing. DSC data confirmed a decrease in the glass transition temperature (Tg) and altered crystallisation behaviour in double-processed samples, consistent with improved interfacial compatibility. Contact angle results showed broadly similar surface wettability across all series, pointing to processing history, rather than surface chemistry, as the key variable governing final mechanical behaviour in these blends.

## 1. Introduction

Growing pressure to replace fossil-based packaging has pushed bio-based and biodegradable polymers to the forefront of materials research. Polylactic acid (PLA) stands out in this field thanks to its renewable feedstock, industrial compostability, and mechanical strength that can rival conventional plastics [1,2]. Its main weakness, however, is brittleness: elongation at break typically falls in the 2–8% range, and fracture occurs at low strain with little plastic deformation. This is a particular obstacle for flexible films: converting, printing and sealing operations impose strains well above the yield point of neat PLA, so toughening is a precondition for use rather than an incremental refinement [3,4].

Two copolyesters have attracted considerable attention as toughening agents for PLA: poly(butylene adipate-co-terephthalate) (PBAT) and poly(butylene succinate) (PBS). PBAT is a fully biodegradable, highly flexible polymer widely used in compostable film production, and its blending with PLA has been shown to yield synergistic improvements in elongation at break [5,6]. The underlying mechanism is well established: a finely dispersed rubbery phase promotes interfacial debonding and cavitation, which relieves triaxial stress and allows the PLA matrix to deform by shear yielding instead of crazing [7,8]. Recent studies have confirmed the strong influence of blend ratio and processing method on the resulting morphology and mechanical response of PLA/PBAT systems [9,10]. PBS, while less flexible than PBAT, offers superior thermal stability and is fully bio-derivable from succinic acid, making it an attractive partner for PLA in food packaging applications [11,12]. Because this mechanism operates only where the dispersed phase is fine and well adhered, immiscibility is the central obstacle: PLA is thermodynamically immiscible with both PBS and PBAT, and the resulting phase-separated blends have a dispersed-phase morphology, and hence a mechanical response, that depends strongly on the preparation method [13,14]. Where interfacial adhesion is poor, coarse droplets act as defects rather than as stress concentrators, and the expected ductility gain is not realised [7].

Strategies for controlling this morphology fall into three groups. The first is chemical. Reactive extrusion using chain extenders, peroxide initiators, or epoxy-functional copolymers can create block or graft copolymers in situ [15,16,17], and is demonstrably effective, but it introduces a reactive additive with an associated cost and, for food-contact film, a regulatory burden [18]. The second group is physical modification of the matrix. The addition of low-molecular-weight plasticizers, particularly poly(ethylene glycol) (PEG), has been shown to reduce Tg, improve chain mobility, and act as a processing aid that indirectly enhances interphase adhesion [19,20]. PEG molecular weight is a critical parameter: short chains (e.g., PEG 400–4000) preferentially plasticize the PLA matrix, while longer chains exhibit more complex partitioning behaviour [21,22,23]. A recent comprehensive study of PLA/PEG 4000 blends confirmed miscibility at moderate loadings with a Tg depression exceeding 19 °C, but also revealed a structured melt state at higher concentrations [23]. Plasticization is nonetheless bounded by migration and physical ageing: PEG-plasticized PLA is known to phase-separate and to lose part of its ductility during storage, and any migrating species must itself be approved for food contact [24,25,26]. The third route is purely processing-based. Multiple melt processing passes, reprocessing, have been reported to alter crystallinity, double melting behaviour, and dispersed phase morphology in PLA-based blends, though the net effect on mechanical performance depends strongly on the blend system [27,28]. That literature has, however, been built almost entirely around mechanical recycling, in which five to seven successive cycles are applied, and the dominant outcome is chain scission and progressive loss of properties [29]. The first additional pass, where morphological refinement should be greatest and thermal-mechanical degradation least, is rarely reported in isolation. Whether that single pass, applied deliberately as a dispersion-refinement step rather than as recycling, can improve morphology enough to outweigh the accompanying degradation has therefore received far less attention than the recycling question it is usually subsumed into.

Direct comparisons of single-pass blending, PEG-assisted processing, and reprocessing within a single experimental framework are rarely reported. Yet this is precisely the comparison required by practitioners who must weigh processing complexity against target properties. To fill that gap, we prepared PLA/PBAT and PLA/PBS blends across four series: single-processed blends, PEG 4000-modified, PEG 20000-modified, and double-processed, and characterised the resulting thin films by tensile testing, DSC, and contact angle measurements. The three techniques answer complementary questions: tensile testing quantifies the ductility gain, DSC establishes whether that gain originates in the crystalline phase or in matrix mobility, and contact angle probes the film surface, which governs printability, sealing and wettability in packaging use [30]. In the double-processed series, the compounded blend was passed through the extruder once more, so the comparison drawn here is between one and two melt-processing passes, not between successive recycling cycles. The specific objectives were therefore (i) to quantify the ductility achievable in each series against a common, additive-free single-pass reference, (ii) to determine whether PEG molecular weight shifts the balance between plasticization and phase separation, and (iii) to establish whether a single additional pass outperforms both the single-pass blend and the PEG-modified series. We hypothesised that it would: that the refinement of dispersed-phase morphology achieved during the second pass would more than offset the chain scission incurred by the additional thermal-mechanical history, giving a route to ductility that is mechanistically distinct from plasticization and more effective than it. None of the systems studied is reactive, so all are physical blends in the broad sense; the term “physical blend” is used here only to designate the single-pass, additive-free reference series. More broadly, the work supports the push toward bio-based alternatives to conventional petrochemical films (SDG 12 and SDG 13) and demonstrates that ductility gains comparable to those achieved with additives can be obtained through a simple, additive-free processing step, a finding with direct relevance to circular bioeconomy manufacturing (SDG 9).

## 2. Materials and Methods

### 2.1. Materials

Polylactic acid (PLA, Ingeo 2003D, Nature Works, Plymouth, MN, USA) was used as the matrix polymer. The number average molecular weight of Ingeo 2003D was 100,422 g/mol, its weight average molecular weight was 180,477 g/mol, and its polydispersity index was 1.79 [31]. Poly(butylene adipate-co-terephthalate) (PBAT, NaturePlast, Mondeville, France) and poly(butylene succinate) (PBS, NaturePlast, Mondeville, France) were used as the flexible polyester components. According to the suppliers’ technical data sheets, the PBAT grade has a density of 1.26 g/cm^3^ and a melt flow index of 4–6 g/10 min (190 C, 2.16 kg), and the PBS grade a density of 1.26 g/cm^3^ and a melt flow index of 22 g/10 min (190 C, 2.16 kg). Poly(ethylene glycol) with nominal molecular weights of 4000 g/mol (PEG 4000) and 20,000 g/mol (PEG 20000) were obtained from Sigma-Aldrich (St. Louis, MO, USA) and Merck (Darmstadt, Germany), respectively. All materials were dried prior to processing according to the manufacturer’s recommendations.

### 2.2. Sample Preparation

Four series of films were prepared:

♦ Series 1: Single-processed blends: PLA was melt-blended with PBS or PBAT at concentrations of 10, 20, and 30 wt% of the flexible component using a custom-built single-screw extruder (Azurr-Technology, s.r.o., Dolní Bečva, Czech Republic) with a barrel temperature profile of 160/170/180 °C, a screw speed of 100 rpm, and a calendering speed of 3 m/min.
♦ Series 2: PEG-modified PLA/PBS blends: PEG 4000 or PEG 20000 was added to PLA/PBS blends at 1, 3, and 5 wt% during melt blending under the same conditions as Series 1.
♦ Series 3: PEG-modified PLA/PBAT blends: As Series 2 but with PBAT as the flexible component.
♦ Series 4: Double-processed blends: PLA/PBAT and PLA/PBS blends (10, 20, 30 wt% flexible component) were subjected to a second melt processing pass under identical conditions, without any additives.

### 2.3. Mechanical Testing

Tensile properties (maximum stress, elongation at maximum stress, tensile strength and elongation at break) were measured according to ISO 527/ASTM D638 [32] using a Shimadzu EZ-LX universal tensile testing machine (Shimadzu, Kyoto, Japan) at a crosshead speed of 10 mm/min. At least five specimens per sample were tested. One film was produced per composition, and at least five specimens were cut at random positions from that single film. The reported means and standard deviations therefore describe within-film, specimen-to-specimen variability rather than batch-to-batch reproducibility. Results are reported as mean ± standard deviation.

### 2.4. Differential Scanning Calorimetry (DSC)

DSC measurements were performed using a TA Instruments Q20 (New Castle, DE, USA) under a nitrogen atmosphere. Samples of approximately 5–10 mg were heated from 0 to 200 °C at 10 °C/min, held for 2 min, then cooled and reheated. The glass transition temperature (Tg), cold crystallisation temperature (Tc), enthalpy of cold crystallisation (ΔHc), melting temperature (Tm) and enthalpy of melting (ΔHm) were recorded. All values reported in Section 3.4 were taken from the first heating scan, so that the thermal history imparted by processing was retained. The degree of crystallinity (Xc) was calculated according to:

Xc (%) = [(Hm − Hc)/(Hm° × wPLA)] × 100

where Hm° = 93.6 J/g is the theoretical enthalpy of melting for 100% crystalline PLA, and wPLA is the weight fraction of PLA in the blend.

### 2.5. Contact Angle Measurements

Static water contact angles and surface tension were measured on film surfaces using the sessile drop method with an Ossila goniometer (Ossila Ltd., Sheffield, UK). At least three measurements per sample were performed and averaged.

### 2.6. Fourier Transform Infrared Spectroscopy (FTIR)

**Figure 1:**
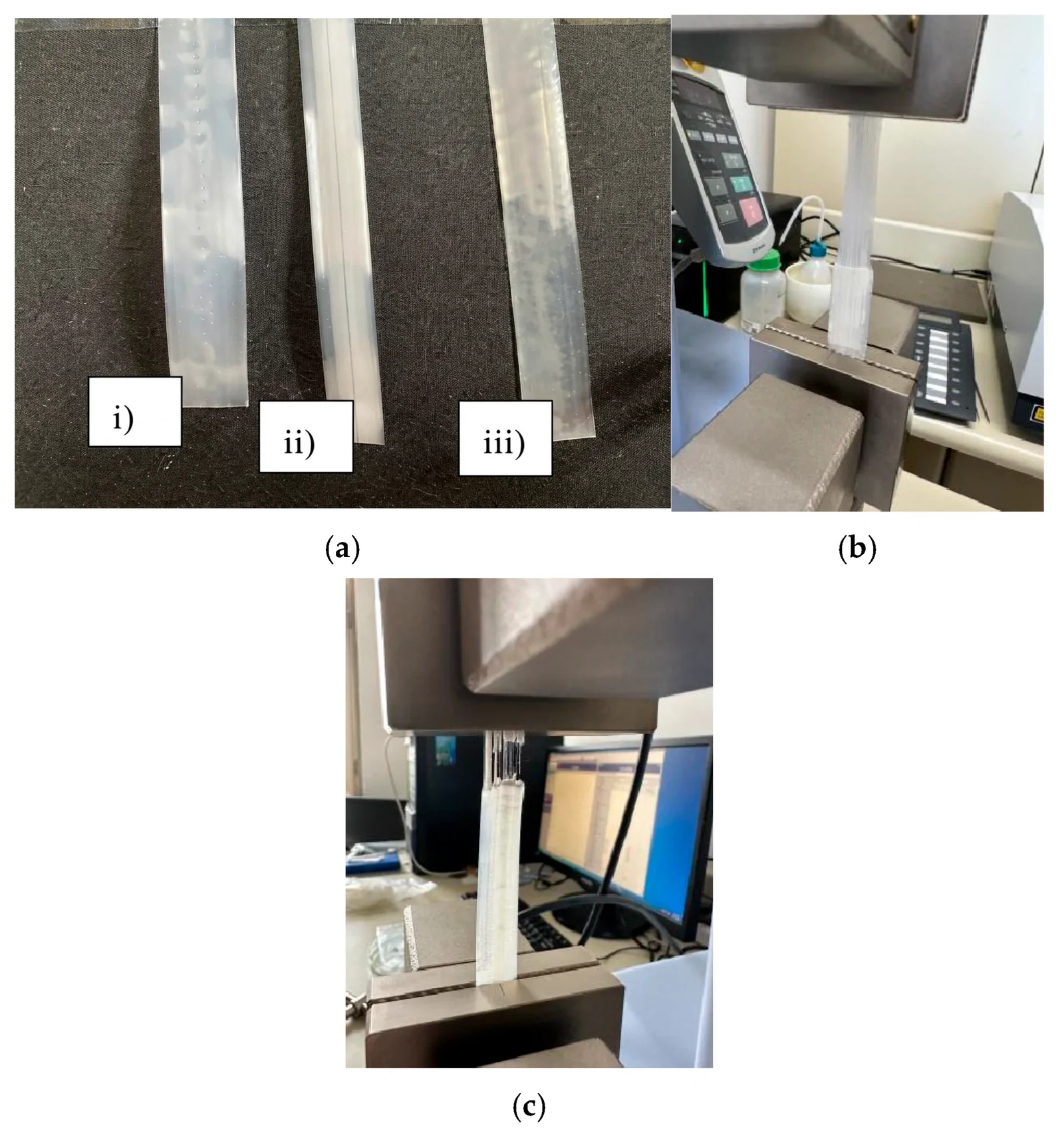
Samples before (**a**) (i) double processed 90PLA/10PBS, (ii) 70PLA/30PBS/5PEG20000, (iii) 90PLA/10PBAT/5PEG20000 and after tensile testing (**b**) double processed 90PLA/10PBS and (**c**) 70PLA/30PBS/5PEG20000.

FTIR spectra were recorded on a Shimadzu IRAffinity-1S spectrometer (Shimadzu, Kyoto, Japan) in the wavenumber range of 400–4000 cm^−1^ with a resolution of 4 cm^−1^. Measurements were performed in ATR/transmission mode at room temperature. Spectra were collected for neat PLA, neat PBAT, neat PBS, and for the 70PLA/30PBAT and 70PLA/30PBS blends after tensile testing. The films were homogeneous after preparation; the spectra were recorded after tensile testing because the deformed specimens developed visually distinct zones along the gauge length (a drawn region, an undrawn region and a second drawn region, see Figure 1b), and we wished to establish whether this optical inhomogeneity was accompanied by any chemical change. Reference spectra of the undeformed films were also collected and were indistinguishable from those of the deformed specimens.

### 2.7. Scanning Electron Microscopy (SEM)

Surface morphology of selected blend films was examined by scanning electron microscopy (SEM) using an Apreo 2C (Thermo Fisher Scientific, Brno, Czech Republic) at an accelerating voltage of 1 kV. Only the free film surfaces were imaged; fracture surfaces were not examined. Because of the low accelerating voltage of 1 kV, the specimens were imaged uncoated, and no conductive sputter coating was applied. All micrographs were recorded at 1000× magnification.

### 2.8. Statistical Analysis

All tensile data were analysed statistically. Two-way analysis of variance (ANOVA) was used to test the effects of blend ratio and of PEG molecular weight (for the PEG-modified series), and of blend ratio and flexible-polyester type (for the additive-free and double-processed series), together with their interaction, on maximum stress, elongation at maximum stress, tensile strength and elongation at break. Pairwise comparisons between single- and double-processed counterparts, and between processing technologies, were made using two-tailed Student *t*-tests. Effect sizes are reported as partial eta squared for ANOVA factors and as Cohen’s d for pairwise comparisons. Differences were considered significant at *p* < 0.05, and are denoted in the tables as * *p* < 0.05, ** *p* < 0.01 and *** *p* < 0.001; ns denotes *p* ≥ 0.05.

## 3. Results

### 3.1. Mechanical Properties of Single-Processed Blends

The tensile properties of the single-pass blended PLA/PBAT and PLA/PBS samples (without any additives) are summarised in Table 1. Pure PLA-based films exhibited characteristic brittle behaviour. The addition of PBAT at 10 wt% yielded a maximum stress of 36.2 ± 6.1 MPa with an elongation of 3.6 ± 1.2%, while increasing PBAT content to 30 wt% reduced tensile strength to 30.4 ± 5.7 MPa while marginally increasing elongation to 4.1 ± 1.6%. The PLA/PBS blend (80/20) showed a maximum stress of 26.7 ± 2.9 MPa and notably lower elongation (2.5 ± 0.09%). These results confirm the expected stiffness-toughness trade-off and set the baseline against which the modified series can be judged. It should be emphasised that the elongation values discussed above, and reported in the fourth column of Table 1, are elongations at maximum stress. The corresponding elongations at break for the same single-processed films were considerably larger—6.88% for 90PLA/10PBAT, 16.88% for 70PLA/30PBAT and 7.16% for 80PLA/20PBS—and are reported in Table 1.

### 3.2. Mechanical Properties of PEG-Modified Blends

The incorporation of PEG 4000 and PEG 20000 into PLA/PBS and PLA/PBAT blends produced varied but generally moderate effects on tensile properties. Selected results for both systems are presented in Table 2. For PLA/PBS blends with PEG 4000, maximum stress values ranged from 20.8 to 29.3 MPa depending on blend ratio and PEG content, with elongations at maximum stress of 3.1–4.8%. The highest tensile stress in this series (29.3 MPa) was observed for the 90PLA/10PBS blend with 3% PEG 4000. With PEG 20000, the 90PLA/10PBS + 1% PEG system achieved a stress of 29.95 ± 1.5 MPa and elongation of 5.6 ± 2.85%, representing one of the better outcomes in the PEG-modified series.

For PLA/PBAT blends, the 90PLA/10PBAT + 1% PEG 4000 blend achieved the highest stress of 31.1 ± 1.3 MPa with elongation at maximum stress of 4.76 ± 1.2%. The 80PLA/20PBAT + 1% PEG 20000 system similarly reached 31.2 ± 2.1 MPa. In general, neither PEG molecular weight nor concentration showed a consistent monotonic trend across all blend ratios, suggesting complex interactions between plasticizer partitioning and the immiscible blend morphology. Elongation at maximum stress in the PEG-modified series rarely exceeded 6%, indicating that plasticization alone does not overcome the fundamental immiscibility of the system.

### 3.3. Mechanical Properties of Double-Processed Blends

Double processing had a pronounced and highly distinctive effect on the mechanical behaviour of the blends, particularly for the PBAT-containing systems. The results are presented in Table 3. The most striking result was observed for the 70PLA/30PBAT blend subjected to double processing, which achieved an average elongation at break of approximately 137%, compared to 16.9% for the single-processed equivalent. Elongation at max stress (typically 3–5%) is the strain recorded at the point of maximum stress, i.e., at yield, whereas “elongation at break” (up to about 137%) is the strain at final fracture. Both are extracted by the testing software from different points of the same stress–strain curve, so the values are complementary rather than contradictory: the double-processed material reaches its stress maximum at low strain and then draws plastically to a much larger strain before failing. Maximum stress was 20.8 ± 2.1 MPa lower than the single-pass blend (30.4 MPa) but accompanied by a complete transition from brittle to ductile fracture behaviour (Figure 1). The 70PLA/30PBS blend also showed substantially elevated elongation at break (~98%) after double processing, with a maximum stress of 26.1 ± 1.7 MPa.

For blends with lower flexible component content, the effect was lower but still significant. The 90PLA/10PBS (2x) blend displayed an average break elongation of 17.3% and the highest maximum stress in the series (38.9 ± 3.6 MPa), suggesting that at low PBS contents, double processing can improve interfacial adhesion without much cost to stiffness—making it the standout approach across all four strategies tested.

### 3.4. Thermal Properties (DSC)

DSC results for the double-processed blends and selected reference samples are presented in Table 4. Neat PLA exhibited a Tg of 62.2 °C, a cold crystallisation exotherm at 93.9 °C (Hc = 13.8 J/g), and a single melting endotherm at 152.9 °C with a crystallinity of 3.1%. All double-processed blends showed a depression of Tg to approximately 55–60 °C, consistent with partial plasticization or an increase in chain mobility at the PLA/polyester interface arising from improved blending.

The double-processed PLA/PBAT blends exhibited double melting peaks (Tm_1_ ~148–150 °C, Tm_2_ ~154–155 °C), a feature that is well documented for PLA [33,34] but which cannot be attributed to a single cause. Two mechanisms are generally invoked, and they are not mutually exclusive: solid-state alpha-prime to alpha reorganisation, in which conformationally disordered alpha-prime crystals formed during processing transform into more stable alpha crystals during heating; and melt-recrystallisation, in which a population of less perfect crystals melts, and the released segments recrystallise into a more stable form before the second melting event. At the single heating rate of 10 C/min used here, the relative contributions of the two mechanisms cannot be separated, which would require variable-rate DSC or temperature-resolved WAXD. Notably, the crystallinity values (Xc) for PLA/PBAT double-processed blends were near zero (apparent negative values, which have no physical meaning, have been set to ~0), indicating that the extended cold crystallisation enthalpy (Hc) approached or exceeded the melting enthalpy, a sign of a predominantly amorphous PLA phase in which crystallisation is promoted by the second processing step. In contrast, the 70PLA/30PBS (2x) blend showed an elevated Xc of approximately 32.96%, with a complex dual melting behaviour at 113.8 and 149.7/154.6 °C, suggesting coexistence of PLA and PBS crystalline phases. The cold-crystallisation temperatures recorded here (107–110 °C, Table 4) fall below the ~120 °C boundary above which the α form grows preferentially, so an α′-rich population is expected on heating, making the α′→α route a plausible contributor to the observed double endotherm [34,35]. Since α′ has lower density and modulus than α, the polymorphic composition is not thermally inconsequential but bears directly on the mechanical response [35].

### 3.5. Contact Angle and Surface Properties

Contact angle measurements showed that all blend series, single-processed blends, PEG-modified, and double-processed, exhibited water contact angles broadly in the range of 65–85°, indicating a moderately hydrophobic surface character consistent with PLA-dominated surface composition. No systematic trend was observed as a function of PBAT or PBS content across any of the series. Surface tension values calculated from contact angle data showed considerable scatter (standard deviations frequently > 100 mN/m), which may reflect surface heterogeneity at the micron scale arising from phase-separated morphology.

The contact angle data for double-processed blends (e.g., 70PLA/30PBAT (2x): 72.9 ± 11.4°; 70PLA/30PBS (2x): 67.8 ± 9.8°) were comparable to those of the physically blended counterparts (70PLA/30PBAT: 71.0 ± 9.5°; 70PLA/30PBS: 77.95 ± 2.6°), confirming that the dramatic improvement in elongation at break observed after double processing does not arise from changes in surface chemistry but rather from bulk morphological changes.

### 3.6. FTIR Analysis

**Figure 2:**
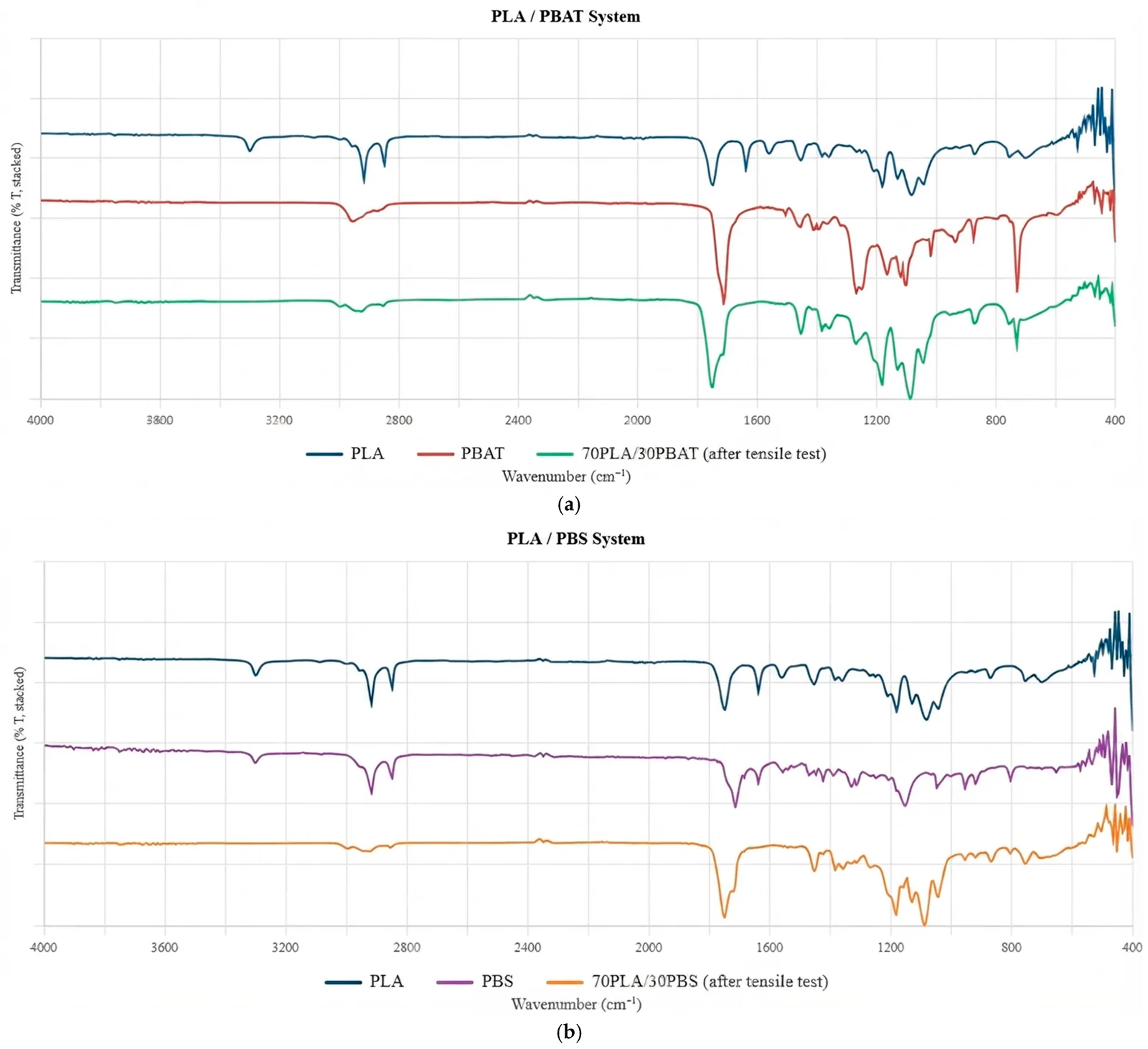
FTIR spectra of (**a**) PLA/PBAT system and (**b**) PLA/PBS system after reprocessing.

FTIR spectra of the neat components and selected blend films are presented in Figure 2. The spectrum of neat PLA showed the characteristic strong carbonyl stretching band (C=O) at approximately 1750 cm^−1^, C–H asymmetric and symmetric stretching bands in the region 2995–2945 cm^−1^, and a complex fingerprint region dominated by C–O–C stretching vibrations at 1267 cm^−1^ and 1087 cm^−1^. Neat PBAT displayed its C=O ester stretch at approximately 1714 cm^−1^, shifted to a lower wavenumber relative to PLA due to the aromatic ring influence, along with a characteristic CH_2_ rocking vibration at ~730 cm^−1^ associated with the butylene segments. Neat PBS exhibited a C=O band at ~1716 cm^−1^, C–O–C stretching at ~1150 cm^−1^, and a CH_2_ rocking mode at ~916 cm^−1^.

The spectra of the 70PLA/30PBAT and 70PLA/30PBS blends after tensile testing showed absorption bands attributable to both constituent polymers, with no new peaks or significant band shifts relative to the neat component spectra. The retention of the PLA carbonyl band position at ~1750 cm^−1^ in both blend spectra indicates that no transesterification or other chemical reaction occurred between the components during melt processing or mechanical deformation. This confirms that the blend systems remain physically mixed, and that the improvement in elongation at break observed for the double-processed samples arises from morphological changes, specifically, refinement of the dispersed phase, rather than from any covalent interfacial bonding.

### 3.7. Statistical Analysis of Mechanical Data

Two-way ANOVA was applied separately to the additive-free, PEG-modified and double-processed series. In the additive-free series, blend ratio significantly affected maximum stress (F = 4.71, *p* = 0.031, partial eta squared = 0.44) and tensile strength (F = 7.51, *p* = 0.008, partial eta squared = 0.56), whereas the identity of the flexible polyester (PBS versus PBAT) was not significant for any property (*p* > 0.33). In the PEG-modified series, blend ratio again dominated: for PLA/PBS it was highly significant for maximum stress (F = 10.20, *p* = 0.0002) and tensile strength (F = 8.67, *p* = 0.0006), and for PLA/PBAT for maximum stress (F = 9.13, *p* = 0.0004). PEG molecular weight was not a significant factor for maximum stress, elongation at maximum stress or elongation at break in either system (*p* > 0.06), and reached significance only for the tensile strength of the PLA/PBAT blends (F = 8.04, *p* = 0.0067), where a significant blend ratio x PEG molecular weight interaction was also detected (F = 6.02, *p* = 0.0046).

For the double-processed series, blend ratio was highly significant for maximum stress (F = 14.58, *p* = 0.0006, partial eta squared = 0.71) and significant for elongation at break (F = 5.97, *p* = 0.016, partial eta squared = 0.50), and polymer type was highly significant for maximum stress (F = 22.33, *p* = 0.0005). Direct single-versus-double comparisons by *t*-test confirmed that reprocessing significantly increased elongation at break for 80PLA/20PBAT (+98%, *p* = 0.004, d = 4.77), 80PLA/20PBS (+175%, *p* = 0.027, d = 2.78) and 90PLA/10PBAT (+278%, *p* = 0.020, d = 3.07). The very large increases recorded for 70PLA/30PBAT (+712%) and 70PLA/30PBS (+1201%) did not reach formal significance (*p* = 0.153 and *p* = 0.088, respectively) because of the correspondingly large scatter in the ductile specimens, although the effect sizes remained large (d = 1.44 and 1.83). Reprocessing also significantly reduced tensile strength for 90PLA/10PBAT (−88%, *p* = 0.001), 90PLA/10PBS (−77%, *p* = 0.005) and 80PLA/20PBAT (−52%, *p* = 0.042), quantifying the strength-ductility trade-off. Pairwise comparison of the processing technologies showed that double processing gave significantly higher maximum stress than single processing with PEG 4000 (*p* = 0.0006, d = 0.85) but significantly lower tensile strength than either PEG series (*p* = 0.001 and *p* < 0.001).

### 3.8. Surface Morphology

**Figure 3:**
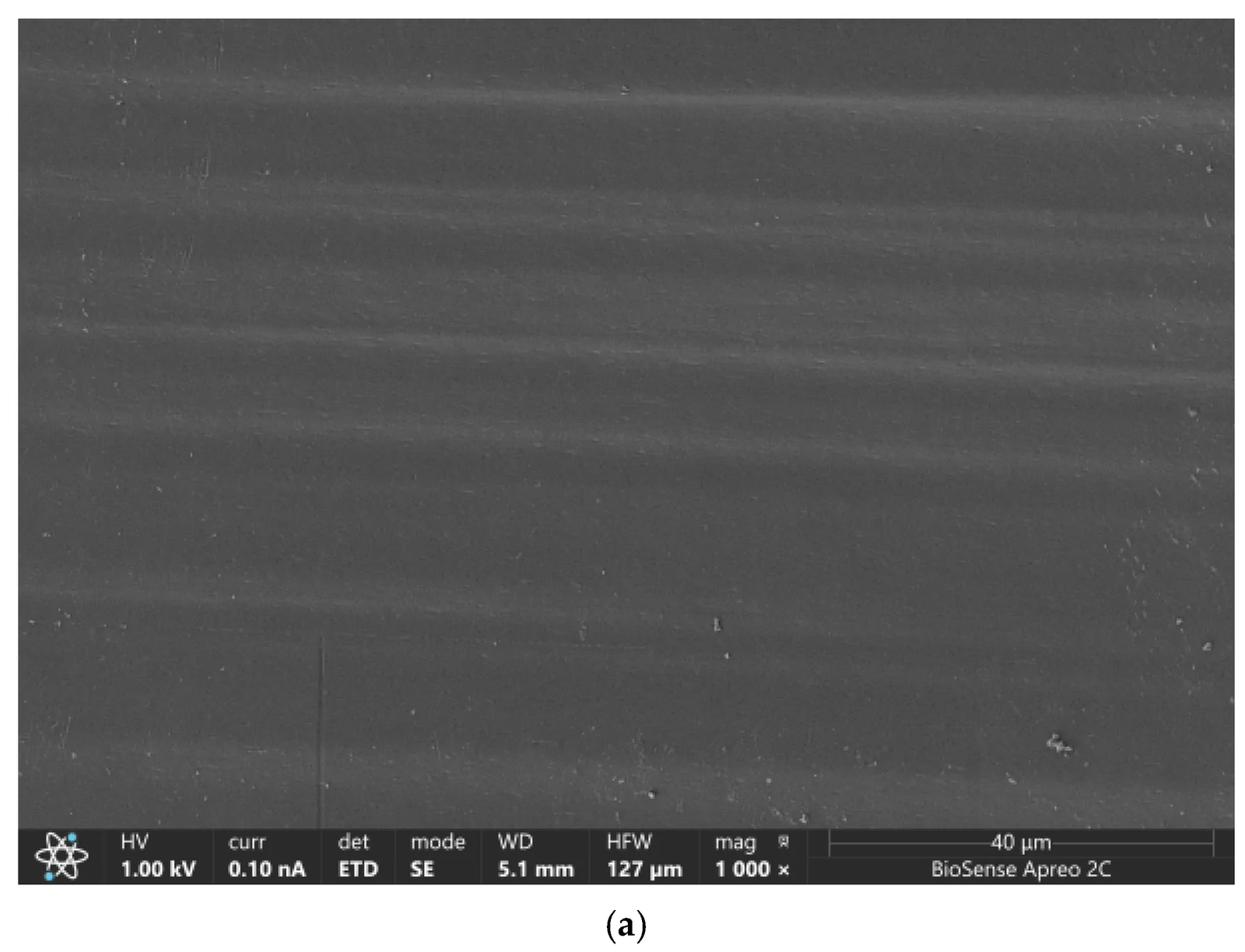

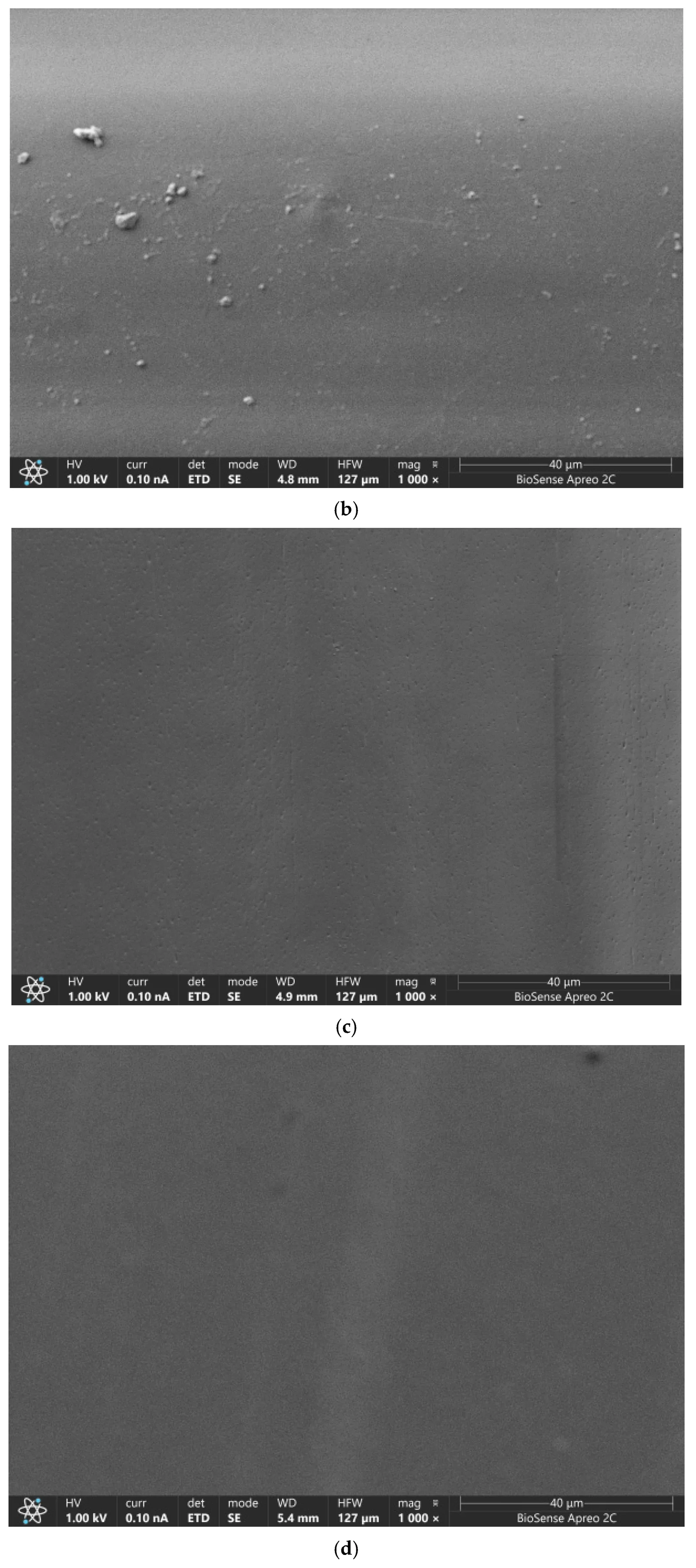
SEM images (**a**) 70PLA/30PBAT, (**b**) 70PLA/30PBS, (**c**) 70PLA/30PBS/3PEG4000, (**d**) 70PLA/30PBAT/1PEG20000.

Although the mechanical behaviour clearly indicates phase separation between the PLA and the PBS or PBAT phase, the SEM images provide no evidence of it. Discrete droplets, or larger separated domains of the flexible polyester, were expected to be visible, but, as Figure 3 illustrates, no such features were resolved on the film surfaces. The likely reason is discussed in Section 4.

## 4. Discussion

The principal finding of this study is that the processing route applied to a PLA-based blend governs its mechanical performance at least as strongly as its composition. Among the four strategies tested, double processing produced the largest gains in ductility by a wide margin, turning brittle blends into materials capable of stretching beyond 100%, without any chemical modification. This is of practical significance, since reprocessing requires no additives and no changes to standard melt processing equipment.

Why does a second processing pass make such a difference? The most likely explanation is morphological refinement. Each pass applies additional shear that progressively breaks up the dispersed flexible phase (PBAT or PBS) into finer droplets and narrows the particle size distribution [27,28]. Finer and more homogeneous dispersion of the rubbery phase promotes stress transfer at the interface and enables greater energy absorption under tensile loading, consistent with the classic rubber toughening mechanism described by Wu for nylon/rubber blends [34]. The DSC data support this interpretation: the depression of Tg in double-processed samples (55–60 °C vs. 62 °C for neat PLA) suggests enhanced chain mobility and increased intermolecular interactions at the PLA/polyester interface, indicative of improved phase compatibility even in the absence of a chemical compatibiliser. This observation aligns with recent findings on reprocessed PBAT/PLA blends, where sequential extrusion passes were shown to influence crystallinity, cold crystallisation behaviour, and double melting peak formation [28].

The near-zero or slightly negative crystallinity values in the double-processed PLA/PBAT blends deserve a closer look. Negative apparent Xc values arise when the cold crystallisation enthalpy exceeds the melting enthalpy, indicating a highly disordered PLA phase in which the second processing step has suppressed crystallisation, a pattern also reported for PLA/PBAT blends subjected to multiple extrusion cycles [28]. For the 70PLA/30PBAT (2x) blend that showed the highest elongation (~137%), the essentially amorphous PLA matrix would permit greater molecular mobility under stress, which, together with the finely dispersed PBAT phase, is consistent with the observed ductile behaviour [20]. The PLA/PBS double-processed system showed a different thermal profile, retaining higher crystallinity (~32.96% for 70PLA/30PBS (2x)) and a dual melting behaviour reflecting PBS crystallinity. The correspondingly lower but still substantial elongation (~98%) reflects the contribution of both phases to deformation, in agreement with literature on compatibilised PLA/PBS systems [14,17].

It is worth separating two crystallisation phenomena that are easily confused. The second extrusion pass suppresses crystallisation during cooling from the melt, so the double-processed films start from a more amorphous state; that same amorphous starting state then promotes cold crystallisation during the subsequent DSC heating scan, which is why the cold-crystallisation exotherm is more pronounced. The two statements are therefore consistent rather than contradictory, and the terms “crystallisation during melt cooling” and “cold crystallisation during DSC heating” are used.

An alternative explanation for the observed behaviour must be considered explicitly: thermomechanical degradation. PLA is known to undergo chain scission during repeated melt processing through a combination of thermal degradation, residual-moisture hydrolysis and shear-induced scission, and a reduction in molecular weight would also increase chain mobility and depress Tg. The Tg depression reported here therefore cannot by itself be taken as proof of improved compatibility. Three arguments nevertheless suggest that chain scission is not the primary origin of the ductility gain. First, the reported thresholds for property-impairing degradation of PLA lie well above two passes: mechanical properties have been reported to remain essentially unchanged up to the third reprocessing cycle, with a significant loss of ductility only beyond the fourth [36], and our own earlier study of the same processing line showed no statistically significant change in Tg, Tc, Tm or tensile strength even after ten consecutive additive-free cycles, with the melt flow index rising only from 8.7 to 13.9 g/10 min over those ten passes [36]. Second, the drivers of severe scission—moisture, melt temperatures above 200 °C and long residence times [37]—were controlled here by pre-drying and by a maximum barrel temperature of 180 °C Third, and most importantly, the expected mechanical signature of chain scission is the opposite of what we observe: shorter chains crystallise more readily and embrittle the material, reducing elongation at break, whereas we record an eight-fold increase for 70PLA/30PBAT (16.9% to 137.1%) and a thirteen-fold increase for 70PLA/30PBS (7.5% to 98.1%). We therefore conclude that although a modest degree of chain scission cannot be excluded in the absence of GPC or melt-flow data, it is unlikely to account for the observed brittle-to-ductile transition. Direct molecular weight determination on the reprocessed films is nevertheless identified as a priority for future work.

Visual inspection of the blend films after tensile testing revealed evidence of phase separation, which provides additional insight into the interfacial characteristics of the PLA/PBAT and PLA/PBS systems. In immiscible polymer blends, tensile deformation subjects the interface between the matrix and dispersed phase to significant stress concentration. When interfacial adhesion is insufficient to sustain stress transfer, debonding of the dispersed phase particles from the matrix occurs, manifesting macroscopically as whitening, voiding, or visible phase separation at or near the fracture zone. This phenomenon, commonly referred to as cavitation or interfacial debonding, is well documented in rubber-toughened thermoplastics and is consistent with the absence of chemical bonding between phases confirmed by FTIR analysis. The observation of phase separation after tensile testing therefore corroborates the physically mixed, immiscible nature of the blends and suggests that while double processing improves the distribution and size of the dispersed phase, enabling greater elongation before failure, it does not fundamentally alter the thermodynamic incompatibility between PLA and the flexible polyester component. This has implications for long-term mechanical durability: under cyclic or prolonged loading, interfacial debonding may initiate premature failure, and future work incorporating reactive compatibilisation or targeted interfacial modifiers could address this limitation while preserving the processing simplicity demonstrated here. The intended application, flexible packaging film, is dominated by short-duration, low-amplitude loading during converting, filling and handling, rather than by sustained cyclic loading, so interfacial debonding is less critical than it would be in a structural component. Nonetheless, creep under sustained tension and fatigue during repeated flexing are relevant to reel handling and to bag or pouch service life, and neither was measured here. Because the blends remain thermodynamically immiscible, progressive debonding under prolonged or repeated loading is a plausible failure route, and cyclic and creep testing of the double-processed films is recommended before industrial adoption.

PEG addition produced more modest and less predictable changes. The lack of a consistent trend with PEG concentration or molecular weight suggests that phase separation between PEG and the PLA/polyester matrix is composition-dependent and that PEG may migrate or crystallise during film formation depending on molecular weight. Recent work on PLA/PEG 4000 blends confirmed that miscibility is maintained up to approximately 20 wt% PEG, beyond which phase separation and crystallisation of PEG occur, leading to complex non-linear property changes [23]. PEG 20000, being a semi-crystalline polymer at room temperature, may partially crystallise within the blend and act as a filler rather than a plasticizer at certain concentrations, explaining the non-monotonic response observed here [22]. While some PEG-modified samples achieved competitive tensile stresses (e.g., 31.2 MPa for 80PLA/20PBAT + 1% PEG 20000), elongation at maximum stress stayed below 6% throughout, confirming that PEG at these concentrations cannot overcome the fundamental immiscibility of the blend, a finding consistent with reports that PEG addition to PLA/PBAT systems improves processing and modulus but not elongation to the extent that morphological optimisation can [38].

Contact angle data confirmed that surface hydrophobicity was essentially invariant across all series and blend compositions. This rules out surface-mediated mechanisms as a contributor to the mechanical differences and points unambiguously to bulk morphological changes, dispersed phase size, distribution, and interfacial adhesion as the controlling variables. SEM of fracture surfaces combined with small-angle X-ray scattering (SAXS) would be a natural next step, allowing direct measurement of dispersed phase size and linking it quantitatively to the mechanical response.

The limits of the present morphological evidence should be stated plainly. The SEM images were recorded on as-cast external film surfaces and show no dispersed domains in any series. This is consistent with the skin effect well documented for extruded biopolyester films, in which rapid surface solidification produces a featureless layer that masks the underlying bulk morphology: Su et al. observed exactly this in PBAT/PLA flat films, where cryo-fractured cross-sections revealed dispersed domains 1–8 μm in size while the external surfaces appeared structureless [39]. The absence of surface domains is therefore an absence of confirmation rather than evidence against a dispersed morphology. Accordingly, the mechanism advanced here—that additional shear refines the dispersed phase and thereby improves stress transfer—is presented as a working hypothesis supported by convergent indirect evidence (absence of new chemistry by FTIR, Tg depression by DSC, invariant surface chemistry by contact angle, and a large reproducible ductility gain achieved without additives) rather than as a proven mechanism. Cryo-fractured or impact-fractured cross-sectional SEM, complemented by small-angle X-ray scattering, is required to measure dispersed-phase size directly and is identified as the priority next step.

The FTIR results provide important mechanistic insight into the nature of the PLA/PBAT and PLA/PBS blends. The absence of new absorption bands and the lack of any wavenumber shifts in the carbonyl region (~1750 cm^−1^ for PLA, ~1714–1716 cm^−1^ for PBAT and PBS) in the blend spectra confirm that no transesterification or chemical reaction occurred between the polyester components during melt processing. This is consistent with the relatively mild processing temperatures employed and the absence of a reactive compatibiliser. The blend spectra can be described as a superposition of the individual component spectra, which is characteristic of an immiscible, physically mixed system. Importantly, this finding reinforces the interpretation of the mechanical data: the striking ductility enhancement observed in the double-processed blends, particularly the ~137% elongation at break for 70PLA/30PBAT, cannot be attributed to the formation of interfacial copolymers or chemical bonding between phases. Instead, it must originate from purely physical changes in blend morphology, most likely a reduction in the size and improved distribution of the dispersed PBAT or PBS phase brought about by the additional shear history of the second processing pass. This conclusion is further supported by the contact angle data, which showed no significant surface chemistry changes across processing series.

## 5. Conclusions

This study compared four blending sample series of PLA/PBAT and PLA/PBS thin films, single-pass melt blending, PEG 4000, PEG 20000, and double processing, and the results point clearly in one direction:

- Double processing is the most effective approach for improving ductility of PLA-based polyester blends, yielding elongation at break values of up to ~137% for 70PLA/30PBAT and ~98% for 70PLA/30PBS, far exceeding all other strategies tested.
- PEG 4000 and PEG 20000 addition at 1–5 wt% produced moderate improvements in tensile stress in selected compositions but did not replicate the ductility enhancement of double processing. The response to PEG content was non-monotonic, reflecting complex plasticizer-blend interactions.
- DSC analysis revealed a depression of Tg and altered crystallisation behaviour in double-processed blends, consistent with improved interfacial compatibility and a more amorphous PLA phase that facilitates ductile deformation.
- Contact angle measurements showed comparable surface hydrophobicity across all series, indicating that mechanical differences are governed by bulk morphology rather than surface chemistry. Double processing offers a simple, additive-free, and industrially scalable route to ductile PLA-based films.
- FTIR analysis confirmed that PLA/PBAT and PLA/PBS blends remain physically mixed systems with no evidence of transesterification or interfacial chemical bonding upon melt processing, establishing that the mechanical improvements observed after double processing are physical rather than chemical in origin. On the basis of the convergent but indirect evidence assembled here, morphological refinement of the dispersed phase is proposed as the most probable mechanism; direct confirmation by cryo-fracture SEM and scattering measurements, together with molecular weight determination to exclude a contribution from chain scission, remains necessary and is the subject of ongoing work.

## Figures and Tables

**Table 1:** Tensile properties of single-pass blended PLA/PBAT and PLA/PBS films (without plasticizer).

Sample	Max Stress (MPa)	SD	Elongation at Max Stress (%)	SD	Tensile Strength (MPa)	SD	Elongation at Break (%)	SD
90PLA/10PBAT	36.21	6.14	3.60	1.2	31.8	4.5	6.88	1.54
70PLA/30PBAT	30.36	5.68	4.13	1.56	20.17	4.25	16.88	16.71
80PLA/20PBS	26.70	2.86	2.54	0.09	18.8	5.55	7.16	3.54

**Table 2:** Selected tensile results for PEG-modified PLA/PBS and PLA/PBAT blends.

Sample	Max Stress (MPa)	SD	Elongation at Max Stress (%)	SD	Tensile Strength (MPa)	SD	Elongation at Break (%)	SD
90PLA/10PBS + 1% PEG 4000	25.20	5.40	3.13	0.94	22.24	5.78	11.20	2.51
90PLA/10PBS + 1% PEG 20000	29.96	1.51	5.97	2.86	25.41	1.67	29.55	17.70
80PLA/20PBS + 3% PEG 20000	30.76	2.36	3.6	0.61	19.57	9.28	17.66	8.6
90PLA/10PBAT + 1% PEG 4000	31.12	1.27	4.76	1.18	24.17	1.18	39.80	13.44
80PLA/20PBAT + 1% PEG 20000	31.18	2.14	3.15	0.26	23.07	0.68	28.43	10.34
70PLA/30PBAT + 3% PEG 4000	24.52	1.23	3.27	0.67	15.53	2.15	125.37	88.40

**Table 3:** Tensile properties of double-processed PLA/PBAT and PLA/PBS blends.

Sample	Max Stress (MPa)	SD	Elongation at Max Stress (%)	SD	Tensile Strength (MPa)	SD	Elongation at Break (%)	SD
70PLA/30PBAT (2x)	20.81	2.11	3.25	0.49	16.07	0.49	137.06	116.97
80PLA/20PBAT (2x)	28.87	3.57	3.18	0.37	14.21	7.14	20.63	2.86
90PLA/10PBAT (2x)	27.17	3.34	4.18	0.69	3.77	3.70	26.01	8.67
70PLA/30PBS (2x)	26.08	1.68	4.44	0.50	13.58	1.26	98.10	69.91
80PLA/20PBS (2x)	33.83	4.51	4.73	1.31	13.55	2.50	19.72	5.31
90PLA/10PBS (2x)	38.86	3.61	4.48	0.50	7.73	6.32	17.34	9.94

**Table 4:** DSC parameters of double-processed blends compared to neat PLA and unprocessed reference blends.

Sample	Tg (°C)	Tc (°C)	Hc (J/g)	Tm_1_ (°C)	Tm_2_ (°C)	Hm (J/g)	Xc (%)
PLA neat	62.2	93.9	13.8	152.9	—	16.7	3.1
70PLA/30PBAT (2x)	56.9	110.3	18.7	149.4	154.2	17.3	~0
80PLA/20PBAT (2x)	56.2	107.0	21.5	148.2	154.2	19.4	~0
90PLA/10PBAT (2x)	60.2	107.5	21.0	149.6	155.4	21.2	0.2
70PLA/30PBS (2x)	55.6	—	—	113.8	149.7/154.6	21.6	32.96
80PLA/20PBS (2x)	59.6	72.7	8.6	114.4	152.5	39.96	14.8
90PLA/10PBS (2x)	55.8	108.5	19.3	149.2	154.5	21.8	2.96

## Data Availability

The data presented in this study are available on request from the corresponding author. Present results are project-derived, and might be subject of some IP protection or commercialization.

## Abbreviations

The following abbreviations are used in this manuscript:

PLA	Polylactic acid
PBAT	Poly(butylene adipate-co-terephthalate)
PBS	Poly(butylene succinate)
PEG	Poly(ethylene glycol)
DSC	Differential scanning calorimetry
Tg	Glass transition temperature
Tc	Cold crystallisation temperature
Tm	Melting temperature
Xc	Degree of crystallinity
wt%	Weight percent
